# Hydrogen-Bonding Interactions in Luminescent Quinoline-Triazoles with Dominant 1D Crystals

**DOI:** 10.3390/molecules22101600

**Published:** 2017-09-22

**Authors:** Shi-Qiang Bai, David James Young, T. S. Andy Hor

**Affiliations:** 1 Institute of Materials Research and Engineering, ASTAR (Agency for Science, Technology and Research), 2 Fusionopolis Way, #08-03, Innovis, Singapore 138634, Singapore; 2 Faculty of Science, Health, Education and Engineering, University of the Sunshine Coast, Maroochydore DC, Queensland 4558, Australia; dyoung1@usc.edu.au; 3 Department of Chemistry, The University of Hong Kong, Pokfulam, Hong Kong, China; andyhor@hku.hk

**Keywords:** structural relationship, H-bonding, crystal growth, 1,2,3-triazole, luminescence

## Abstract

Quinoline-triazoles 2-((4-(diethoxymethyl)-1*H*-1,2,3-triazol-1-yl)methyl)quinoline (**1**), 2-((4-(m-tolyl)-1*H*-1,2,3-triazol-1-yl)methyl)quinoline (**2**) and 2-((4-(p-tolyl)-1*H*-1,2,3-triazol-1-yl)methyl)quinoline (**3**) have been prepared with CuAAC click reactions and used as a model series to probe the relationship between lattice H-bonding interaction and crystal direction of growth. Crystals of **1**–**3** are 1D tape and prism shapes that correlate with their intermolecular and solvent 1D lattice H-bonding interactions. All compounds were thermally stable up to about 200 °C and blue-green emissive in solution.

## 1. Introduction

**Scheme 1:**
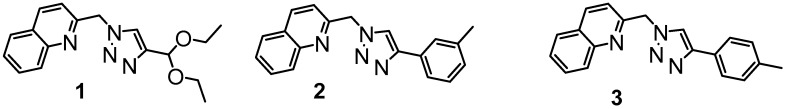
Compounds **1**–**3**.

Molecular growth and design offers the tantalizing prospect of controllable assembly of functional supramolecular materials and inorganic-organic hybrid materials [1,2,3,4,5,6,7,8,9,10]. Organic molecules play important roles in hybrid structures to tune the magnetism, catalytic activity, luminescence, electrical conductivity and thermoelectric effect [11,12,13]. Despite considerable progress in recent years, this field could still be regarded as in its infancy with serendipity playing the major role in breakthrough discoveries. What appears to be lacking is understanding of (and control over) weak inter-molecular forces that fashion the gross structure and function. One fundamentally important tool to elucidate this relationship between molecular form and function in the solid state is single-crystal XRD [14,15,16,17,18,19,20]. A great many organic molecules and metal complexes have been synthesized and structurally characterized by this technique [21,22,23,24,25,26,27,28,29]. The controlled growth of crystals and material assembly, however, is still a challenge because of this lack of correlation between intermolecular forces and bulk morphology. The structural effect of ligands on metal complexes has been investigated extensively and we have contributed to this with, for example, a series of Cu(I) complexes supported by quinoline-triazole ligands bearing different tail groups [30]. Tuning of the ligand structures resulted in different magnetic coordination polymers [31,32]. Mono- and bis-chelating ligands similarly permit tuning of cluster-structures [33]. Less attention, however, has been directed to the relationship between molecular structure and material shape. Our recent study of triazole-pyridine-supported Cu(I) dimers demonstrated that the shape/growth direction of their single crystals correlated with the weak lattice interactions such as hydrogen-bonding and π···π stacking interactions [34]. The dominant plate crystals of Cu(II) complexes also align with their lattice 2D hydrogen-bonding interactions [35]. We have extended this analysis in the current study with an investigation of a series of three quinoline-triazoles (**1–3**) bearing different triazole substituents. (Scheme 1) These compounds were conveniently prepared using CuAAC click chemistry in good to high yields. These hitherto unreported molecules are air stable and luminescent.

## 2. Results and Discussion

### 2.1. Synthesis

**Scheme 2:**
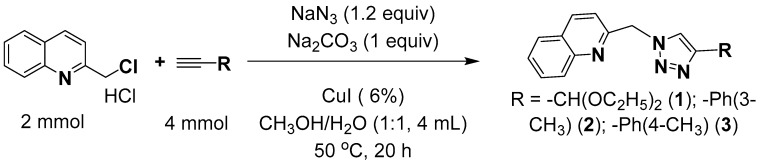
Synthesis of **1**–**3** from CuAAC click reactions.

2-(Chloromethyl)quinoline hydrochloride (428 mg, 2 mmol), Na_2_CO_3_ (210 mg, 2 mmol), NaN_3_ (143 mg, 2.4 mmol), alkyne (4 mmol) and CuI (23 mg, 0.12 mmol) were placed in a reaction tube containing a mixed solvent of CH_3_OH/H_2_O (1:1 *v*:*v*, 4 mL). Each reaction was stirred at 50 °C for 20 h on a MultiMax reactor. The alkynes, 3,3-diethoxy-1-propyne, 3-ethynyltoluene and 4-ethynyltoluene were used for the synthesis of **1**–**3**, respectively. (Scheme 2) On completion, each mixture was extracted with ethyl acetate three times. The combined organic extract was washed with brine, concentrated and purified by column chromatography on silica. 2-((4-(diethoxymethyl)-1*H*-1,2,3-triazol-1-yl)methyl)quinoline (**1**), C_17_H_20_N_4_O_2_, 312.37. Yield: 560 mg, 90%. ESI-MS (*m*/*z*, %): [**1** + H]^+^ (313, 100). Main IR bands (cm^−1^): 3111 (m), 3069 (m), 2976 (m), 2926 (m), 2904 (m), 2878 (m), 1618 (m), 1598 (m), 1567 (m), 1506 (m), 1455 (m), 1426 (m), 1385 (m), 1345 (m), 1320 (m), 1309 (m), 1271 (m), 1225 (m), 1178 (m), 1117 (m), 1097 (m), 1061 (s), 1050 (s), 1038 (m), 1021 (m), 1004 (m), 910 (m), 873 (m), 847 (m), 823 (m), 803 (m), 778 (m), 745 (m), 734 (m), 618 (m), 491 (m), 479 (m) and 432 (m). The bands among 1004–1117 cm^−1^ should be attributed to the C−O stretching vibrations. ^1^H-NMR (CDCl_3_, 500.2 MHz) δ 8.15 and 8.13 (d, 1H, *J* = 9 Hz), 8.07 and 8.05 (d, 1H, *J* = 9 Hz), 7.82 and 7.80 (d, 1H, *J* = 8 Hz), 7.76–7.73 (m, 2H), 7.58–7.55 (m, 1H), 7.28 and 7.27 (d, 1H, *J* = 9 Hz), 5.83 (s, 2H, quinoline-CH_2_), 5.71 (1H, CHO_2_), 3.73–3.56 (m, 4H, CH_2_CH_3_), 1.22, 1.21 and 1.19 (t, 6H, CH_3_). ^13^C-NMR (CDCl_3_, 125.8 MHz) δ 154.6, 147.85, 147.77, 137.8, 130.3, 129.3, 127.8, 127.7, 127.3, 122.8 and 119.7 (quinoline and triazole groups), 96.9, 61.8, 56.5 (quinoline-*C*H_2_-triazole) and 15.2.

2-((4-(*m*-Tolyl)-1*H*-1,2,3-triazol-1-yl)methyl)quinoline (**2**), C_19_H_18_N_4_O (**2**·H_2_O), 318.37. Yield: 510 mg, 80%. ESI-MS (*m*/*z*, %): [**2** + H]^+^ (301, 100). Main IR bands (cm^−1^): 3134 (m), 2944 (m), 2918 (m), 1664 (m), 1615 (m), 1600 (m), 1568 (m), 1508 (m), 1488 (m), 1460 (m), 1432 (m), 1420 (m), 1360 (m), 1322 (m), 1230 (m), 1218 (m), 1179 (m), 1142 (m), 1121 (m), 1082 (m), 1045 (m), 980 (m), 961 (m), 894 (m), 847 (m), 821 (m), 797 (s), 784 (m), 760 (m), 749 (m), 727 (m), 697 (m), 618 (m), 477 (m), 442 (m) and 410 (m). ^1^H-NMR (CDCl_3_, 500.2 MHz) δ 8.17–8.10 (m, 2H), 7.94–7.93 (b, 1H), 7.81–7.79 (m, 1H), 7.77–7.73 (m, 1H), 7.63 (s, 1H), 7.58, 7.56 and 7.55 (t, 2H, *J* = 8 Hz), 7.34–7.30 (m, 1H), 7.25 and 7.23 (d, 1H, *J* = 8 Hz), 7.10 and 7.08 (d, 1H, *J* = 8 Hz), 5.90–5.88 (b, 2H, quinoline-CH_2_) and 2.34 (s, 3H, CH_3_). ^13^C-NMR (CDCl_3_, 125.8 MHz) δ 154.5, 148.6, 147.1, 138.6, 138.5, 130.7, 130.4, 129.1, 128.82, 128.78, 127.9, 127.7, 127.5, 126.5, 123.0, 120.4 and 119.9 (quinoline and triazole groups), 56.0 (quinoline-CH_2_-triazole) and 21.5 (CH_3_).

2-((4-(*p*-Tolyl)-1*H*-1,2,3-triazol-1-yl)methyl)quinoline (**3**), C_19_H_16_N_4_, 300.36. Yield: 490 mg, 82%. ESI-MS (*m*/*z*, %): [**3** + H]^+^ (301, 100). Main IR bands (cm^−1^): 3132 (m), 3111 (m), 2939 (m), 2920 (m), 1617 (m), 1601 (m), 1568 (m), 1499 (m), 1460 (m), 1425 (m), 1356 (m), 1323 (m), 1313 (m), 1225 (m), 1187 (m), 1141 (m), 1118 (m), 1076 (m), 1049 (m), 1022 (m), 976 (m), 890 (m), 821 (m), 812 (s), 778 (m), 763 (m), 746 (m), 730 (m), 616 (m), 517 (m), 477 (m) and 415 (m). ^1^H-NMR (CDCl_3_, 500.2 MHz) δ 8.17 and 8.16 (d, 1H, *J* = 9 Hz), 8.11 and 8.09 (d, 1H, *J* = 9 Hz), 7.89 (s, 1H), 7.84 and 7.82 (d, 1H, *J* = 8 Hz), 7.78–7.75 (m, 1H), 7.71–7.70 (m, 2H), 7.60–7.57 (m, 1H), 7.34 and 7.32 (d, 1H, *J* = 9 Hz), 7.22 and 7.20 (d, 2H, *J* = 8 Hz), 5.88 (s, 2H, quinoline-CH_2_) and 2.36 (s, 3H, CH_3_). ^13^C-NMR (CDCl_3_, 125.8 MHz) δ 154.9, 148.7, 147.8, 138.2, 137.9, 130.4, 129.6, 129.4, 127.9, 127.8, 127.7, 127.3, 125.8, 120.0 and 119.8 (quinoline and triazole groups), 56.6 (quinoline-*C*H_2_-triazole) and 21.4 (CH_3_).

### 2.2. Molecular Structures

**Figure 1:**
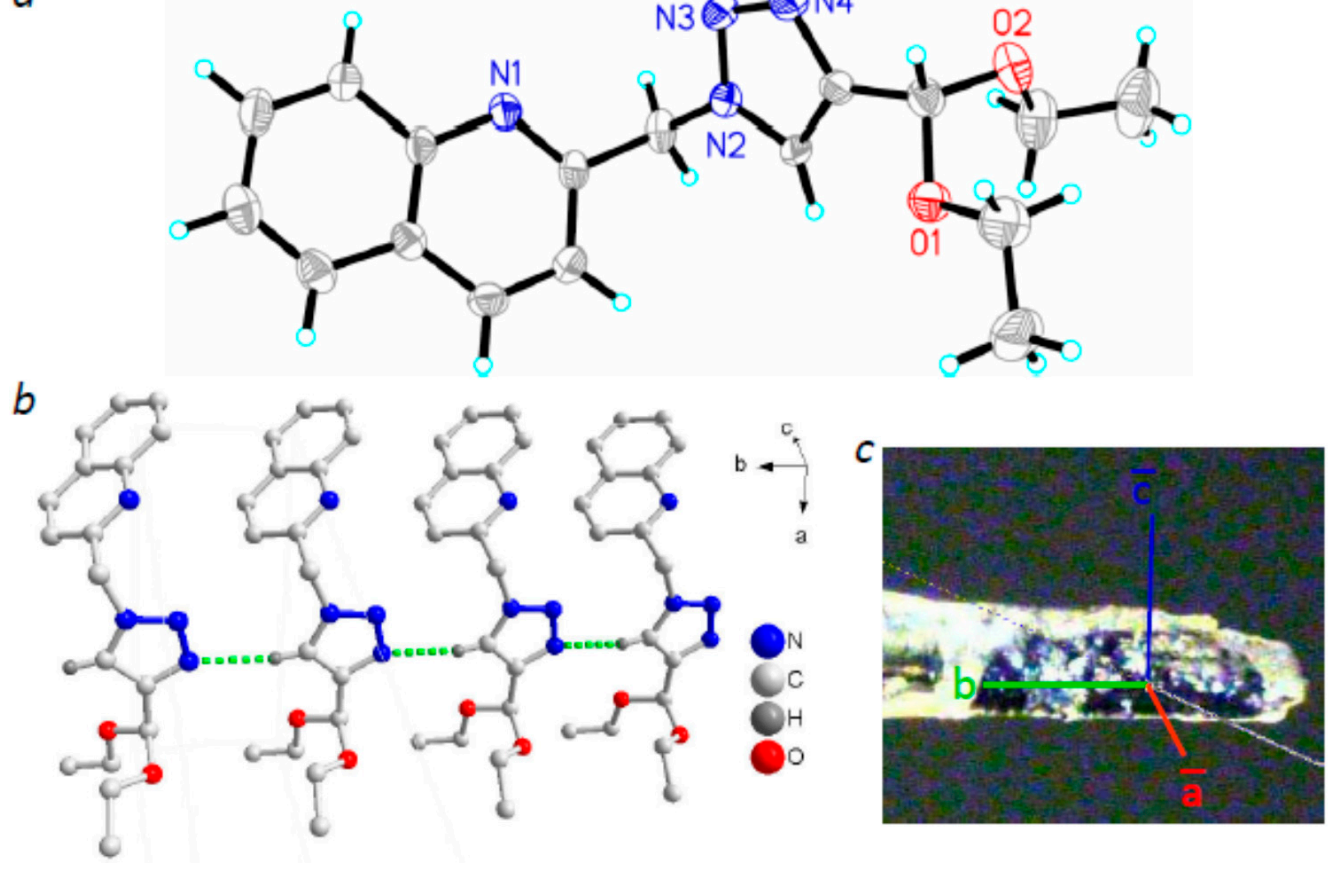
Molecular structure of **1** (**a**). H-bond interactions in **1** (**b**). Crystal image with lattice directions of **1** (**c**).

**Figure 2:**
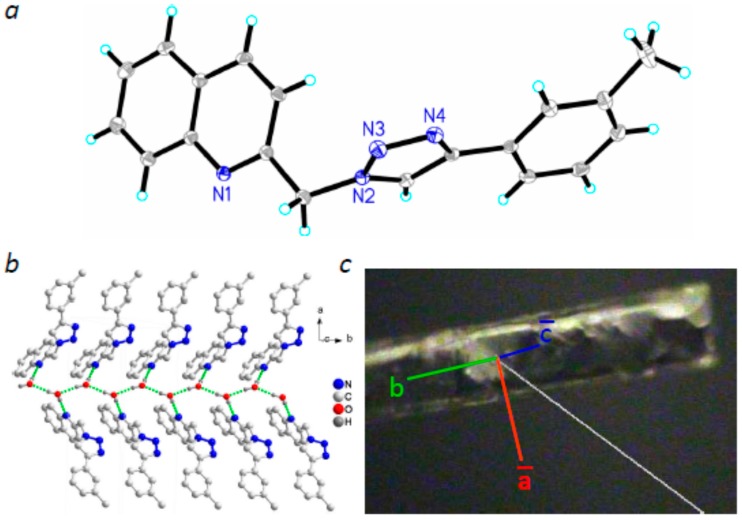
Molecular structure of **2** (**a**). H-bond interactions with lattice water molecules in **2** (**b**). Crystal image with lattice directions of **2** (**c**).

**Figure 3:**
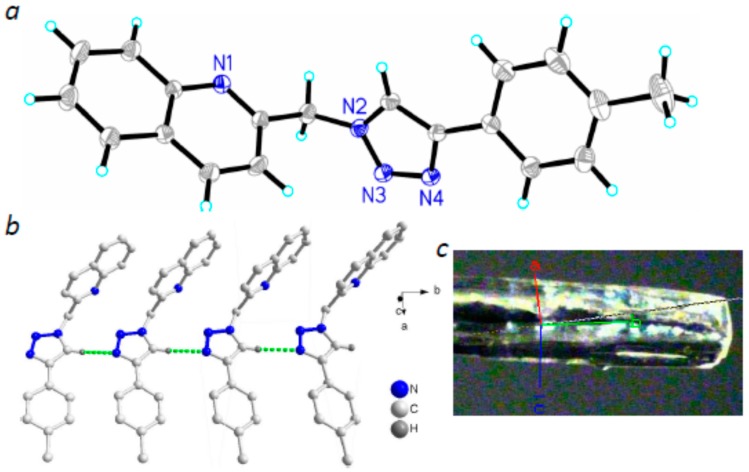
Molecular structure of **3** (**a**); H-bond interactions in **3** (**b**); Crystal image with lattice directions of **3** (**c**).

Quinoline-triazoles **1**–**3** were synthesized using 2-(chloromethyl)quinoline hydrochloride, sodium azide and an alkyne (3,3-diethoxy-1-propyne, 3-ethynyltoluene or 4-ethynyltoluene) in good to high yields (80–90%). The structures of **1**–**3** were characterized by ^1^H-NMR and ^13^C-NMR spectroscopy (Supplementary Materials, Figures S1–S6), electrospray ionization mass spectrometry, single-crystal X-ray crystallography. These compounds crystalized in different dominant shapes, *viz* belt and prism. Compound **2** crystallized in the orthorhombic crystal system with space groups of *P*bca. Compounds **1** and **3** crystallized in the monoclinic crystal system with the same space group of *P*2_1_/*c*. The open and flexible substituent group of **1** has only one obvious H-bonding interaction (C11−H···N4A, Table 1, Figure 1) between the triazole (C11) and 3’-N_tri_ atom along the *b* direction, which is also aligned with the dominant direction in the belt crystal. Molecules **2** and **3** are less flexible analogues of **1**, bearing rigid tolyl groups. The introduction of an m-tolyl group in **2** aligns the molecular packing along the *b* direction and captures water molecules in that same direction. These packing and encapsulation effects contribute to the formation of 1D water H-bonding interactions (O1−H···N1 and O1−H···O1A, Table 1, Figure 2) between the lattice water and quinoline N acceptor, and between neighbouring water molecules along the lattice *b* direction. These results suggest that the backbone H-bonding interactions are disrupted by the introduction of small lattice molecules, in this case H_2_O. The 1D water chains (in the *b* direction) align with the dominant crystal belt direction (*b* axis). Compound **3** bearing a pendant p-tolyl group is isostructural with **2** in generating a prism shaped crystal along the dominant *b* axis. The bulk crystal does not capture lattice solvent, but displays 1D H-bond interactions as in **3**, (Table 1, Figure 3) which similarly drive 1D crystal growth. Intramolecular constraints imposed by a rigid pendant group thus appear to influence H-bonding formation and therefore crystal shape. The C−H···N H-bonding interactions were formed between the neighboring triazole rings along the *b* direction in **1** and **3**, but not in **2**. These triazole rings are parallel to each other in *b* direction in **1**–**3**, their center-center distances are 5.52, 5.23 and 5.45 Å, and plane-plane distances are 0.29, 2.14 and 1.28 Å, respectively. The large plane-plane distance (2.14 Å) in **2** probably decreases the possibility of forming intermolecular H-bonding interaction.

This series of molecules show some correlation between compound lattice structures and the resulting shape of bulk single crystals. The lattice H-bonding directions are aligned with the direction of material growth, which also align with the shortest axis (*b*) in all three compounds **1**–**3**. We are currently investigating if such correlation can be extended to related systems, and the potential of small molecule (e.g., water) entrapment to control crystal shape, size and function. 

### 2.3. Powder XRD, TGA, UV-vis and Photoluminescent Spectroscopy

**Figure 4:**
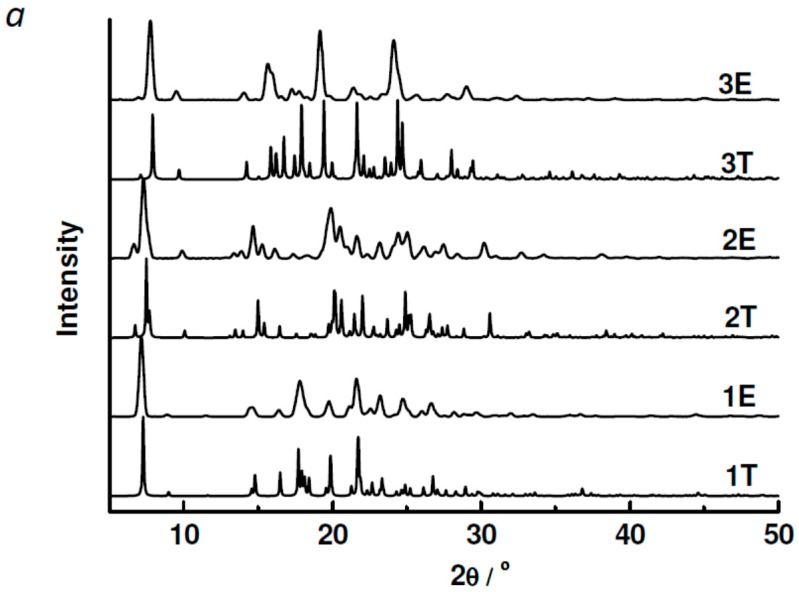

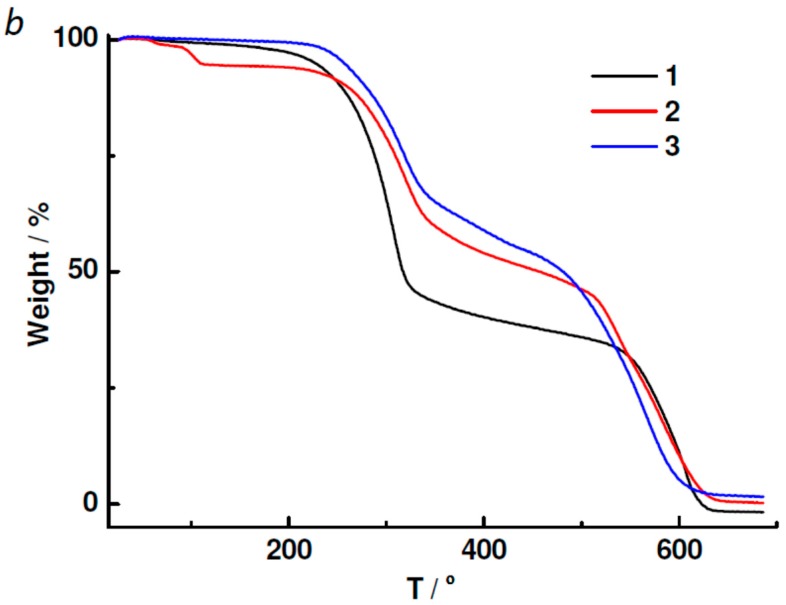
(**a**) Powder XRD patterns of **1**–**3**. (T = theoretical profile referenced to the experimentally determined single-crystal XRD pattern; E = experimental data). (**b**) TGA curves of **1**–**3**.

The experimental powder X-ray diffraction patterns for compounds **1**–**3** showed good agreement with their simulated patterns determined from single-crystal XRD experiments, supporting phase purity (Figure 4a). Thermogravimetric analysis (TGA) curves of **1**–**3** from room temperature to 700 °C are given in Figure 4b. Compounds **1**, **2** and **3** were stable in air to 200, 220 and 220 °C, respectively. The weight loss (~5.3%) before 115 °C in **2** was assigned to the loss of lattice water molecules (5.7%). All three compounds continue to degraded and reach residual weights at about 640 °C.

**Figure 5:**
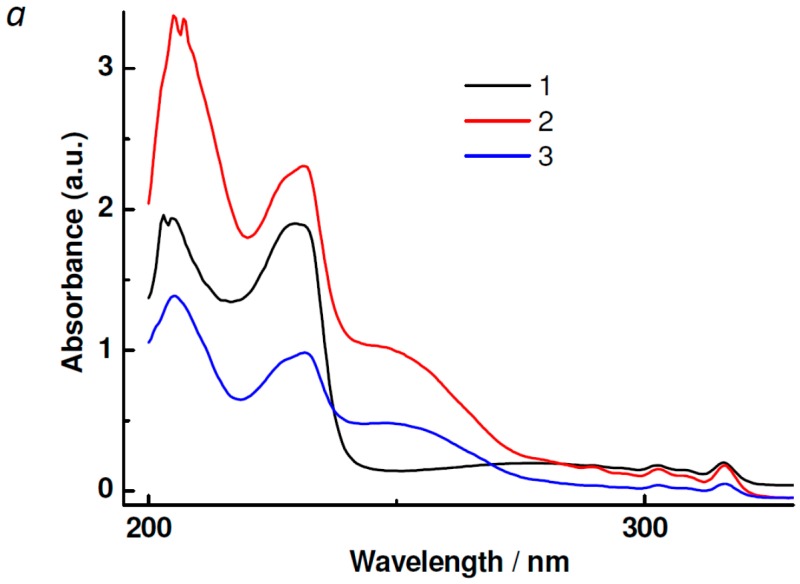

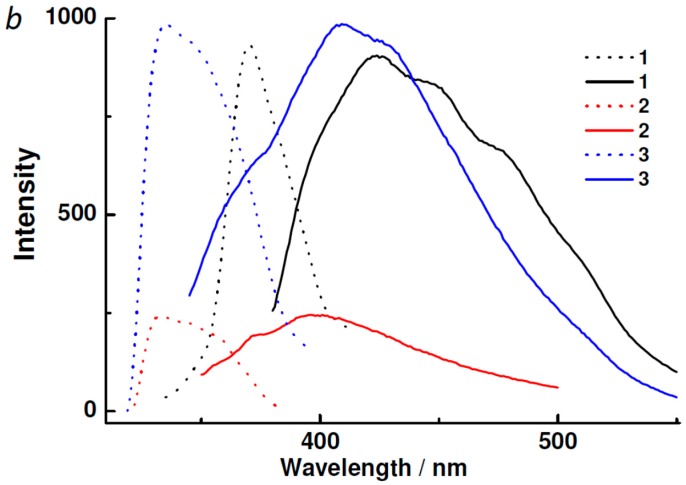
(**a**) UV-vis absorption spectra of **1**–**3** in ethanol solution; (**b**) Excitation (dotted line) and emission (solid lines) spectra of compound **1**–**3** in ethanol solution.

The UV-vis electronic absorption spectra of **1** in ethanol displayed two broad absorption bands centred at 205 and 230 nm (Figure 5a). Compounds **2** and **3** also displayed these absorptions with an additional band at 250 nm that can be attributed to intramolecular charge transfer and π···π interactions of the toluene substituent groups. All solution samples of **1**–**3** were luminescent at room temperature. Compounds **1**–**3** displayed broad emissions centered at 425, 395 and 409 nm respectively with excitation at 370, 334 and 334 nm (Figure 5b). The emissions in the blue-green part of the visible spectrum most likely arise from a π→π* transition. The tolyl substituents gave blue-shifted emissions in **2** and **3** relative to **1** with **3** the strongest emitter.

## 3. Materials and Methods 

All starting chemicals were used as received. Electrospray ionization mass spectra (ESI-MS) were recorded in positive ion mode using a Shimadzu LCMS-IT-TOF mass spectrometer. NMR spectra were measured at room temperature using a JEOL 500 MHz NMR spectrometer. Thermogravimetric analysis (TGA) was carried out in an air stream using a *TA* Instruments TGA Q500 analyzer with a heating rate of 30 °C/min. Photoluminescence spectra were measured on a Shimadzu RF-5301 PC spectrofluorophotometer. UV-vis absorption spectra were recorded on a Shimadzu UV-2501PC UV-Vis recording spectrophotometer. Infrared spectra were obtained on a Perkin Elmer Spectrum 2000 FT-IR spectrometer from samples in KBr disc. Powder X-ray diffraction data were collected on a Bruker (Billerica, MA, USA) AXS GADDS X-ray diffractometer with Cu-Kα radiation (*λ* = 1.54056 Å). Single-crystal X-ray diffraction data were collected using a Bruker AXS SMART APEXII CCD diffractometer using Mo-K_α_ radiation (*λ* = 0.71073 Å) (Table 2). Data integration and scaling were performed using the Bruker SAINT program [36]. Empirical absorption correction was performed by SADABS [37]. The space group determination, structure solution and least-squares refinements on |*F*|^2^ were carried out using Bruker SHELXL [38]. Structures were solved by direct methods to locate the heavy atoms, followed by difference maps for the light non-hydrogen atoms. Anisotropic thermal parameters were refined for the rest of the non-hydrogen atoms. The high R factor (0.0920) in compound **1** could be due to poor quality of its single-crystal. Hydrogen atoms were placed geometrically and refined isotropically. CCDC 1453356(**1**), 1453357(**2** H_2_O) and 1453358(**3**) contain the supplementary crystallographic data for this paper. These data can be obtained free of charge via http://www.ccdc.cam.ac.uk/conts/retrieving.html (or from the CCDC, 12 Union Road, Cambridge CB2 1EZ, UK; Fax: +44 1223 336033; E-mail: deposit@ccdc.cam.ac.uk).

## 4. Conclusions

This work reports a series of new, luminescent quinoline-triazoles **1**–**3**, conveniently prepared by copper-catalyzed azide-alkyne cycloaddition reactions. Lattice H-bonding interactions observed from their single-crystal structures align with the shortest axis. They are also the primary driving force for the direction of bulk crystal growth. This trend was seen for all three compounds and we are investigating the predictive capabilities of this correlation between intermolecular interactions and the direction of crystal growth with a view to more rational crystal engineering.

## Figures and Tables

**Table 1:** Lattice H-bonding interactions in compounds **1**–**3**.

D−H···A	D−H (Å)	D···A (Å)	H···A (Å)	∠D−H···A (^°^)
**1**				
C11−H···N4A	0.93	3.333(4)	2.44	162
Symmetry code A: *x*, *y*−1, *z*.				
		**2**		
O1−H···N1	0.89	2.850(2)	1.96	172
O1−H···O1A	0.84	2.793(2)	1.96	177
Symmetry code A: 0.5−*x*, 0.5+*y*, *z*.				
**3**				
C11−H···N4A	0.95	3.455(2)	2.61	149
Symmetry code A: *x*, 1+*y*, *z*.				

**Table 2:** Summary of crystallographic data for compounds **1**–**3**.

Compound	1	2·H_2_O	3
Formula	C_17_H_20_N_4_O_2_	C_19_H_18_N_4_O	C_19_H_16_N_4_
*M_W_*	312.37	318.37	300.36
*T*/K	298(2)	110(2)	150(2)
Crystal size/mm^3^	0.65 × 0.16 × 0.04	1.10 × 0.30 × 0.05	1.40 × 0.20 × 0.10
Crystal system	Monoclinic	Orthorhombic	Monoclinic
Space group	*P*2_1_/*c*	*P*bca	*P*2_1_/*c*
*a*/Å	12.574(1)	23.617(1)	12.638(1)
*b*/Å	5.5153(4)	5.2305(2)	5.4474(4)
*c*/Å	24.654(2)	26.299(1)	22.694(2)
*α*/°	90	90	90
*β*/°	104.563(2)	90	99.956(2)
*γ*/°	90	90	90
*V*/Å^3^	1654.8(2)	3248.6(2)	1538.8(2)
*Z*	4	8	4
*D*_calc_/g cm^−3^	1.254	1.302	1.297
*μ*/mm^−1^	0.085	0.084	0.080
*θ* range/°	1.67–25.88	1.55–26.42	1.64–26.38
Reflections collected	44361	29756	24940
Independent reflections [*R*_int_]	3182 [0.0522]	3349 [0.0392]	3148 [0.0313]
Parameters	208	225	208
GOF	1.059	1.025	1.038
*R*_1_ (*I* > 2*σ*(I))	0.0920	0.0386	0.0429
*wR*_2_ (all data)	0.2857	0.1108	0.1217

## Supplementary Materials

The Supplementary Materials are available: Figures S1–S6.
