# Supplementary material for: Hydrogen-Bonding Interactions in Luminescent Quinoline-Triazoles with Dominant 1D Crystals
Source: Molecules. 2017 Sep 22;22(10):1600. doi: 10.3390/molecules22101600 (PMC6151808; doi:10.3390/molecules22101600)
Supplement: Supplementary file 1 [file molecules-22-01600-s001.pdf]

molecules

MDPI

Supplementary Materials for:

Hydrogen-Bonding Interactions in Luminescent Quinoline-Triazoles with Dominant 1D Crystals

Shi-Qiang Bai, David James Young and T. S. Andy Hor

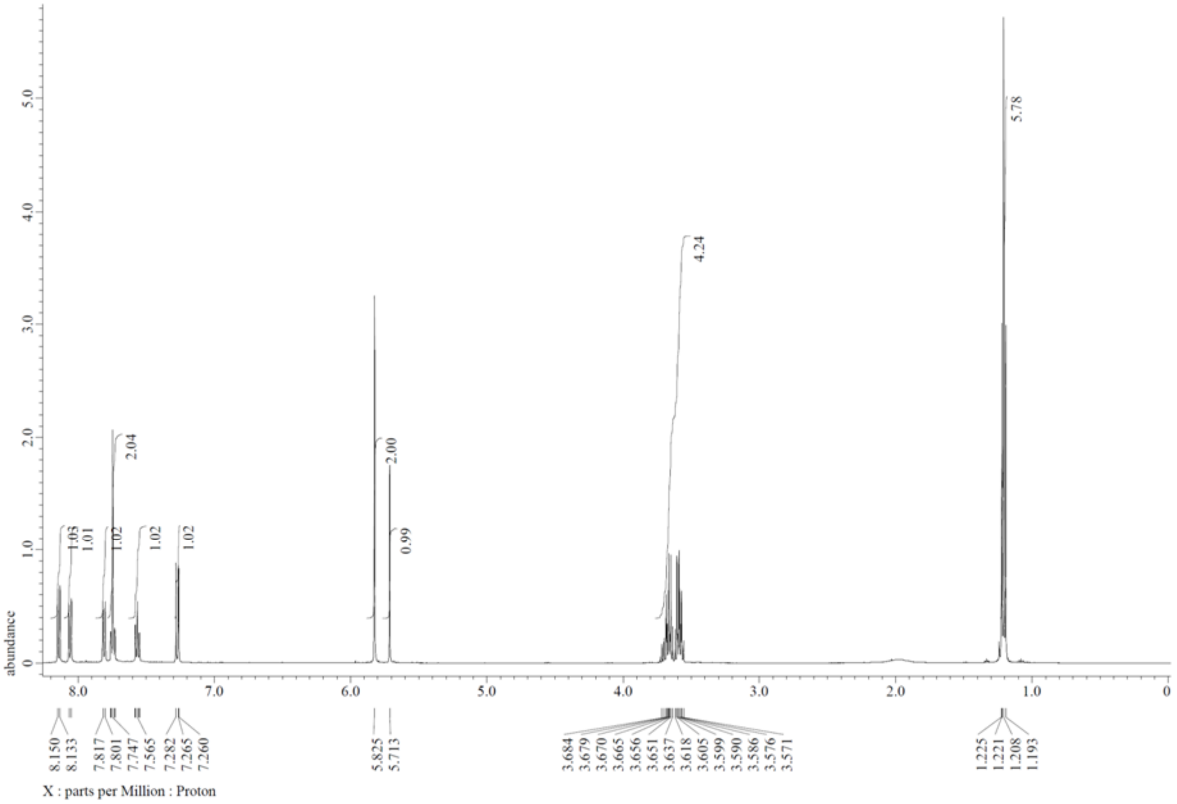

Figure S1. ^{1}H-NMR spectrum of **1**.

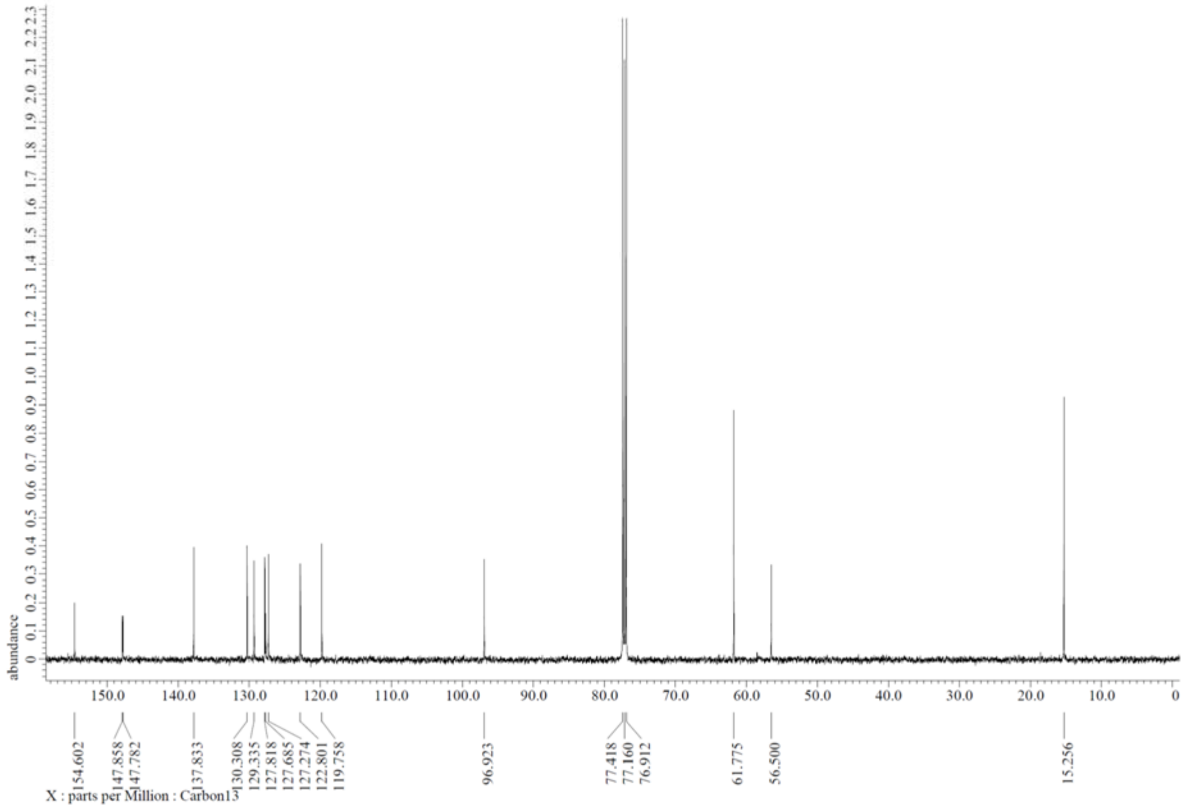

Figure S2. ^{13}C-NMR spectrum of **1**.

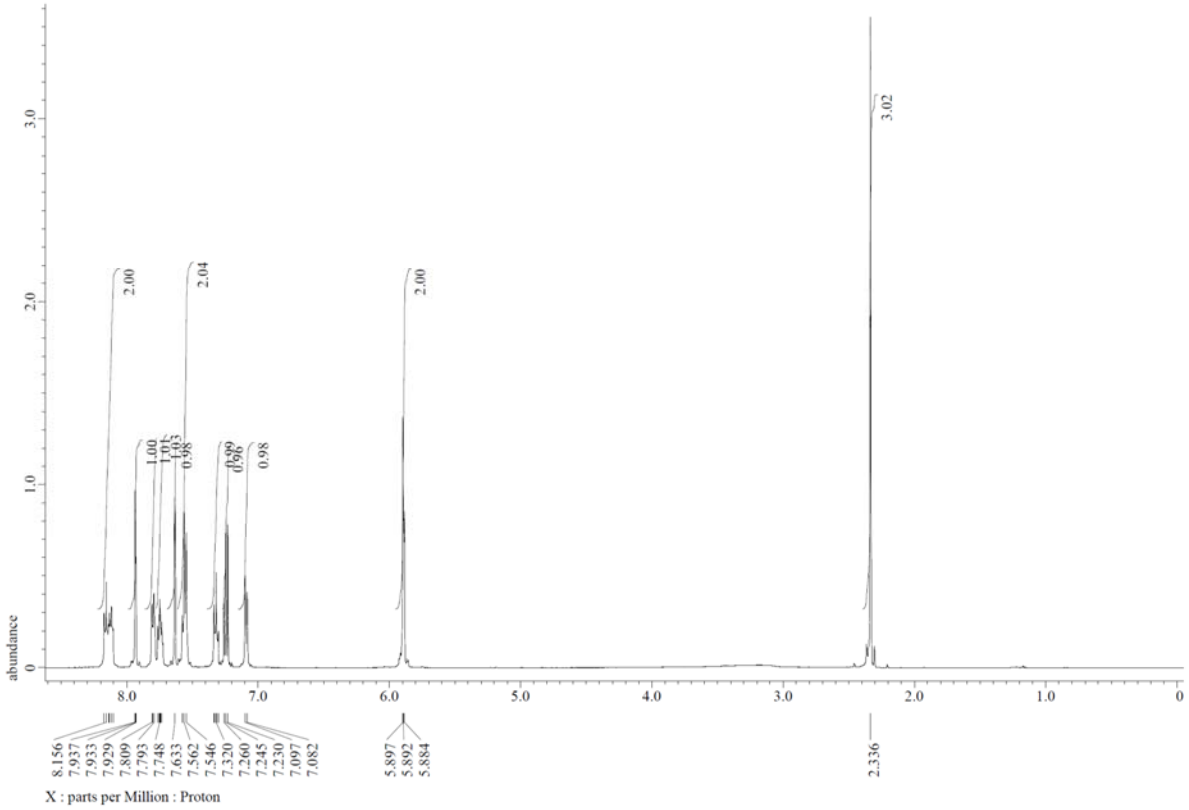

Figure S3. ^{1}H-NMR spectrum of **2**.

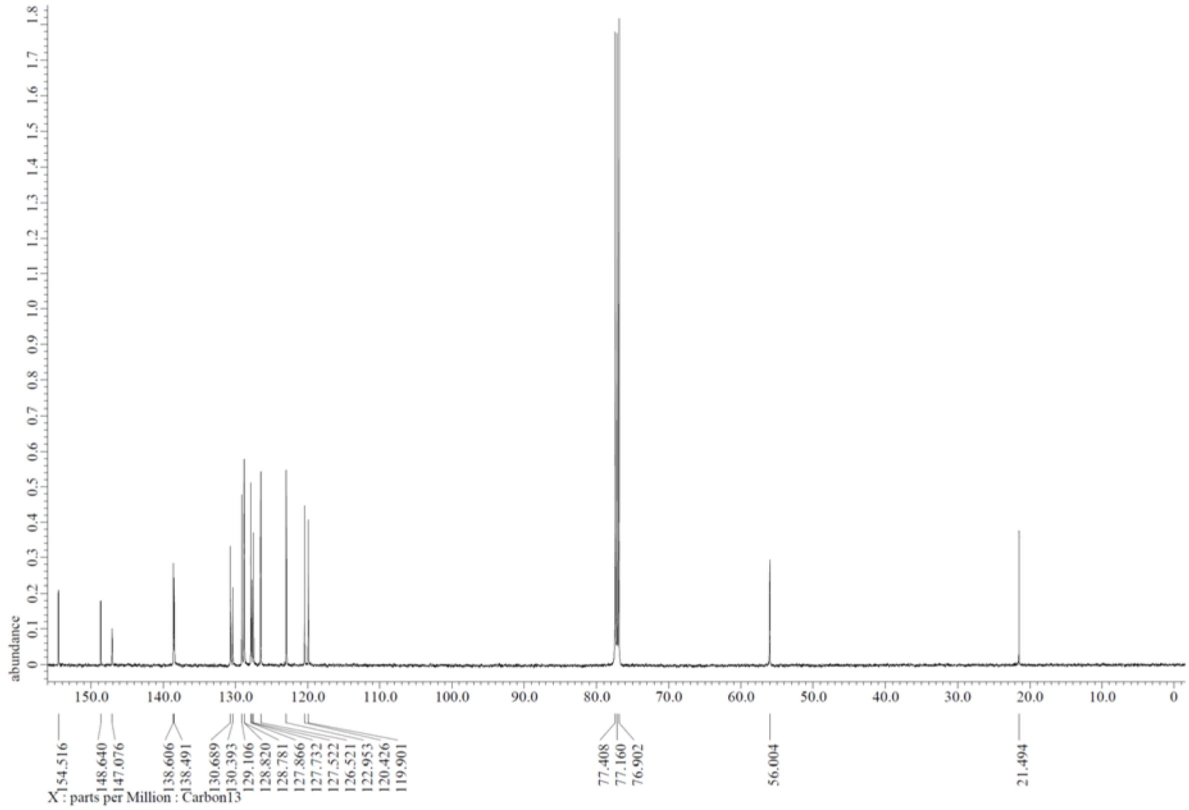

Figure S4. ^{13}C-NMR spectrum of **2**.

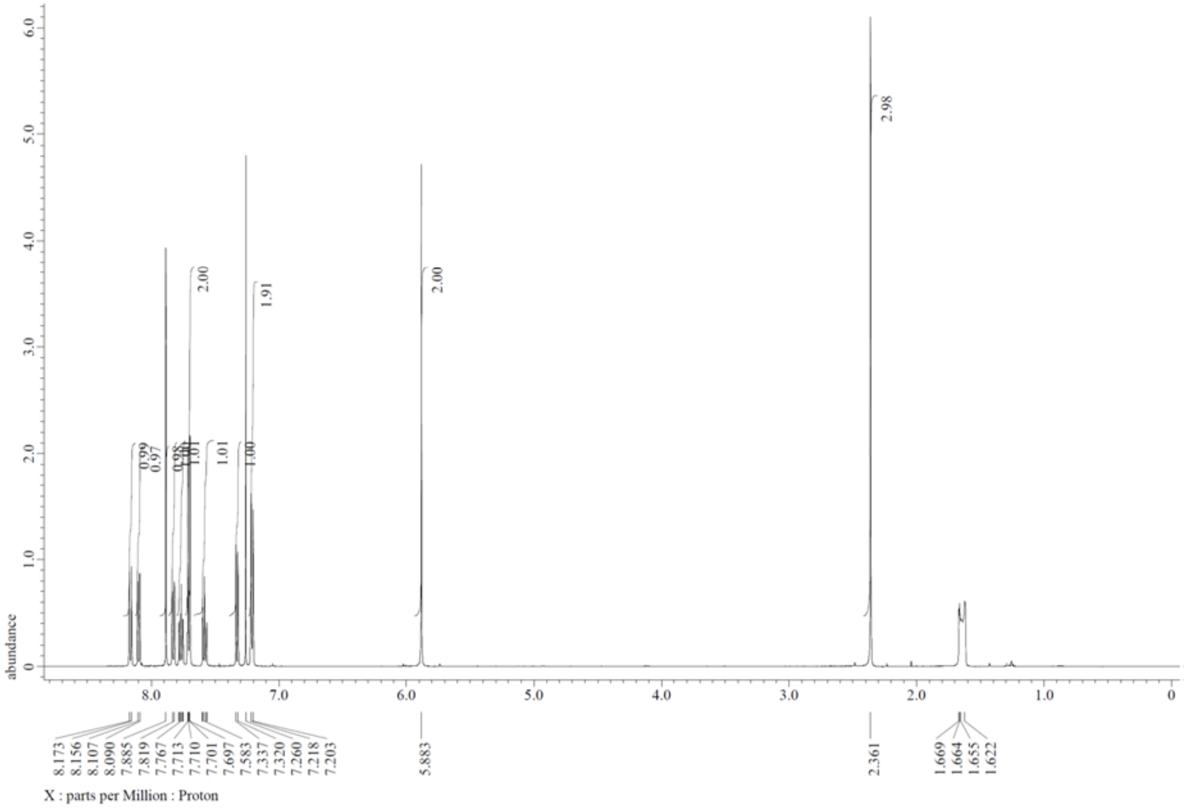

Figure S5. ^{1}H-NMR spectrum of **3**.

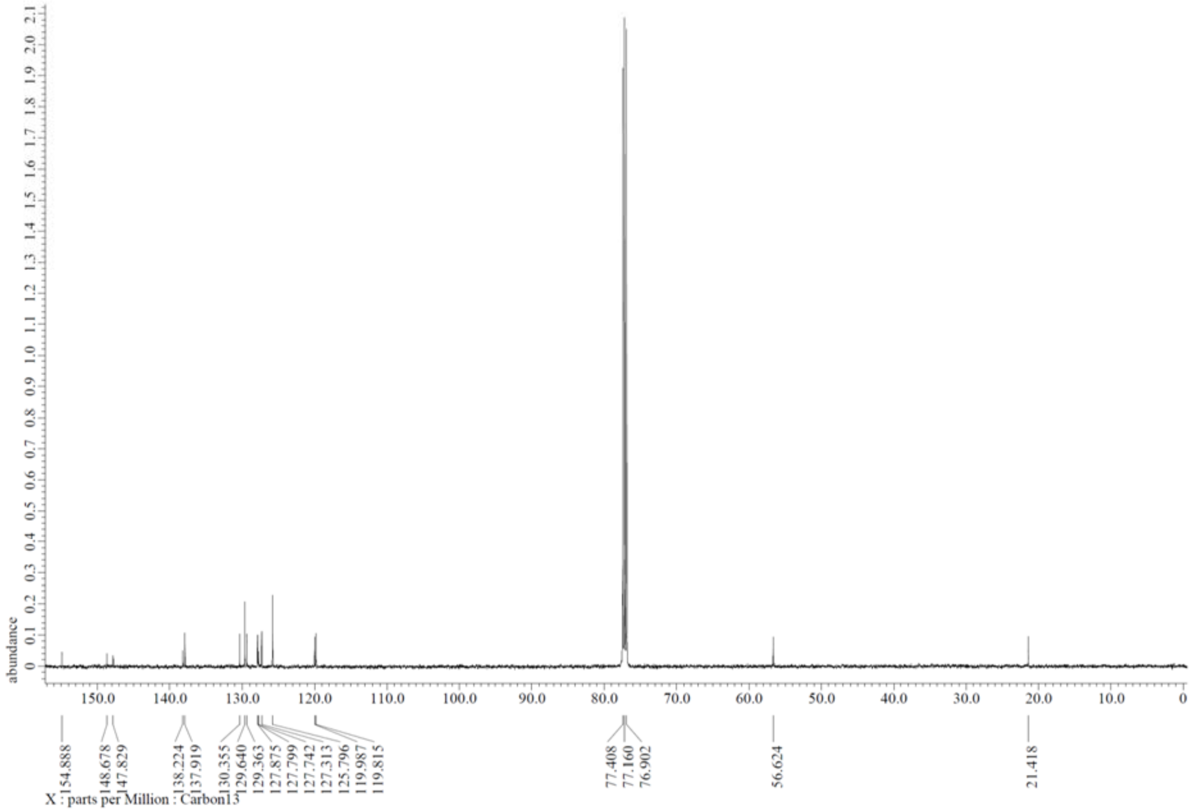

Figure S6. ^{13}C-NMR spectrum of **3**.

© 2017 by the authors. Submitted for possible open access publication under the terms and conditions of the Creative Commons Attribution (CC-BY) license (http://creativecommons.org/licenses/by/4.0/).
